# Antibiotic effectiveness for children with lower respiratory infections: prospective cohort and trial in primary care

**DOI:** 10.3399/BJGP.2022.0239

**Published:** 2022-12-13

**Authors:** Paul Little, Taeko Becque, Alastair D Hay, Nick A Francis, Beth Stuart, Gilly O’Reilly, Natalie Thompson, Kerenza Hood, Michael Moore, Theo Verheij

**Affiliations:** Primary Care Population Sciences and Medical Education Unit, University of Southampton, Southampton, UK.; Primary Care Population Sciences and Medical Education Unit, University of Southampton, Southampton, UK.; Centre for Academic Primary Care, Bristol Medical School: Population Health Sciences, University of Bristol, Bristol, UK.; Primary Care Population Sciences and Medical Education Unit, University of Southampton, Southampton, UK.; Primary Care Population Sciences and Medical Education Unit, University of Southampton, Southampton, UK.; Primary Care Population Sciences and Medical Education Unit, University of Southampton, Southampton, UK.; Primary Care Population Sciences and Medical Education Unit, University of Southampton, Southampton, UK.; Centre for Trials Research, College of Biomedical and Life Sciences, Cardiff University, Cardiff, UK.; Primary Care Population Sciences and Medical Education Unit, University of Southampton, Southampton, UK.; Julius Center for Health Sciences and Primary Care, University Medical Center Utrecht, Utrecht, Netherlands.

**Keywords:** antibiotic resistance, antibiotics, chest infections, children, primary care

## Abstract

**Background:**

Antibiotics are commonly prescribed for children with chest infections but there is little randomised evidence and trials commonly recruit selected populations, which undermines their applicability.

**Aim:**

To document the effectiveness of antibiotics for chest infections in children.

**Design and setting:**

This was a prospective cohort study with nested trial in primary care.

**Method:**

Children aged 1–12 years presenting with uncomplicated lower respiratory tract infections were included in the cohort. Children were either randomised to receive amoxicillin 50 mg/kg per day for 7 days or placebo, or participated in a parallel observational study, where propensity scores controlled for confounding by indication. The outcomes were duration of symptoms rated moderately bad or worse (primary outcome) and illness progression requiring hospital assessment.

**Results:**

A total of 764 children participated (438 trial, 326 observational), and children were more unwell than in previous cohorts (more sputum, fever, shortness of breath). Children had been unwell for a median of 5–6 days, and symptoms rated moderately bad or worse lasted another 6 days when no antibiotics were given.

With antibiotics there was a non-significant reduction of approximately 1 day in duration of symptoms rated moderately bad or worse for the whole cohort (hazard ratio [HR] 1.16, 95% confidence interval [CI] = 0.95 to 1.41), similar to the trial alone (HR 1.13, 95% CI = 0.90 to 1.43). The effect of antibiotic treatment on secondary outcomes was also non-significant.

**Conclusion:**

Antibiotics for uncomplicated chest infections, even in a sample of more unwell children, are unlikely to be clinically very effective.

## INTRODUCTION

Lower respiratory tract infection (LRTI) is a frequent trigger for attendance in primary care — where nearly all children are managed, most still receiving antibiotics.^1^^–^4 Individuals using antibiotics are likely to have more antibiotic-resistant organisms,^5^ which result in prolonged infections subsequently.^6^ Outpatient antibiotic prescribing is linearly related to antimicrobial resistance (AMR).^7^ AMR is a global public health threat^8^^,^^9^ as much of modern medicine (for example, complicated infections, cancer care, surgery) relies on antibiotics.

Clinicians and patients worry about more severe illness, and clinicians often prescribe antibiotics ‘just in case’ for fear of medicolegal consequences,^10^^–^13 and are particularly likely to prescribe for presentations with chest signs, fever, where the individual is judged to be more unwell, sputum/rattly chest, and shortness of breath.^14^^–^17 It is difficult to criticise clinicians’ uncertainty as there are very limited data on the effectiveness of antibiotics for children with chest infections: only two trials in the Cochrane review of antibiotics for acute bronchitis^18^ included children — one trial in patients aged ≥3 years, which included 100 children,^19^ and a small trial (*N* = 140) in those aged ≥8 years.^20^ However, trial data are commonly limited by external validity and substantially greater drug compliance compared with observational studies.^21^^–^23 Conversely, observational data have the disadvantage of confounding by indication (that is, clinicians select individuals for treatment according to the clinical presentation), so techniques are required to control for the propensity to prescribe.^24^^,^^25^ Assuming confounding by indication can be controlled, adding observational data to trial data can increase the power and external validity of analyses, which can help tailor patient information, inform monitoring of disease, or help decisions about treatment.^26^^–^30

The most recent trial in children (ARTIC PC) reported that there was probably limited benefit of antibiotics in children, with symptom resolution happening approximately 1 day sooner, which was not significant.^31^ In the current study, data are reported from the wider cohort of children in the ARTIC PC study, which included both observational data and the nested trial data (amoxicillin versus placebo) using the same measures, and controlling for the propensity to prescribe in the observational data.

## METHOD

### Study design

This was a study of a cohort of children including both observational data and data from a parallel nested randomised placebo-controlled trial.

**Table:** How this fits in

Antibiotics are commonly prescribed for children with chest infections, but prescribing antibiotics fuels antibiotic resistance, which is one of the major global public health threats. There is little randomised evidence and trials commonly recruit selected populations that undermine their applicability. In a cohort of unwell children, antibiotics for chest infections were not effective in significantly shortening the illness and increased side effects. GPs should support parents to self-manage chest infections at home and communicate clearly on when and how to seek medical help if they continue to be concerned.

### Overview of methods

Full details of all data collection methods have been previously published.^31^ Children were recruited between 6 months and 12 years old, presenting to primary care in UK general practices with acute uncomplicated LRTI. Parents and children were invited to participate and consented for participation by the responsible clinicians (usually GPs).

Acute LRTI was defined in several previous cohorts and trials as an acute cough as the predominant symptom, judged by the GP to be infective in origin, lasting <21 days, and with other symptoms or signs localising to the lower respiratory tract (shortness of breath, sputum, pain).^32^^–^34 Exclusion criteria were acute illness requiring immediate referral to hospital (for example, pneumonia, sepsis), non-infective causes of cough (for example, hay fever), and inability to provide consent. These inclusion/exclusion criteria were also used in this study.

Where parents and clinicians were willing for children to be randomised, they were randomised to receive amoxicillin 50 mg/ kg per day in divided doses for 7 days, or placebo, using pre-prepared packs randomised using a computer-generated random number by an independent statistician.^31^ Children not randomised (because ineligible or clinician or parent choice) participated in an observational study where the same baseline clinical data and all outcome data as for the trial were collected by the same methods.^30^ In the observational study, the choice of treatment was at the physician’s discretion and could involve antibiotic prescription or no prescription. Most practices that recruited children to the trial also recruited to the observational study but some sites could only recruit to the observational study.

#### Outcomes

The primary outcome was the duration of symptoms rated moderately bad or worse (a score of ≥3) measured each day using a validated diary^35^ on a seven-point scale (0, normal/not affected; 1, very little problem; 2, slight problem; 3, moderately bad; 4, bad; 5, very bad; 6, as bad as it could be). Secondary outcomes were symptom severity on days 2–4 (0, no problem to 6, as bad as it could be); symptom duration until rated as very little/no problem; primary care re-consultation for new or worsening symptoms (documented by medical record review); side effects (from the diary); and progression of illness (illness requiring hospital assessment and/or admission, within 1 month of the index consultation — documented from a medical record review).^36^

#### Sample size

The study was specifically powered for illness progression — to have sufficient power for the trial sample alone^31^ but to have greater power by including the observational data. To detect a difference in illness duration of 3 days it was estimated it would be necessary to have 119 children in the subgroup with chest signs (alpha 0.05, 80% power) or a total sample of 298 for 80% power. For other subgroups (fever; physician rating of unwell; sputum/rattly chest; short of breath) it was estimated it would be necessary to have 225 children for 90% power and alpha 0.01.

### Statistical analysis

Cox regression was used for the primary outcome, and for total symptom duration, adjusting for age, baseline symptom severity, prior duration of illness, and comorbidity. Linear regression was used for symptom severity, and logistic regression for re-consultation, progression of illness, and side effects, adjusting for the same baseline covariates as in the primary analysis. To aid interpretation, risk ratios were also calculated for the binary outcomes using a log-binomial model. Analysis was by intention to treat (antibiotic group as randomised in the trial sample, and by initial antibiotic treatment in the observational sample) regardless of non-adherence or protocol deviations. Multiple imputation was used as the primary analysis, agreed with the funder. Multiple imputation included all variables from the analysis model and any predictors of missingness (further details are provided in Supplementary Table S1).

The plan had been to control for confounding by indication in the observational dataset by using inverse probability of treatment weighting (IPTW) using propensity scores in each of the regression models. However, the IPTW approach did not achieve good balance on the key covariates, whereas a post hoc analysis using stratification by propensity score did improve balance, and therefore was used in analysing both the observational data and the combined dataset that included both observational and trial data. Participants were stratified based on their propensity to receive antibiotics, with the aim of balancing the covariates between those who receive antibiotics and those who did not within each stratum. The stratum-specific treatment effects were then combined to obtain an overall treatment effect. The propensity scores were calculated separately for the observational and trial data, and regression analyses were then carried out on the pooled sample.

## RESULTS

**Figure 1.**
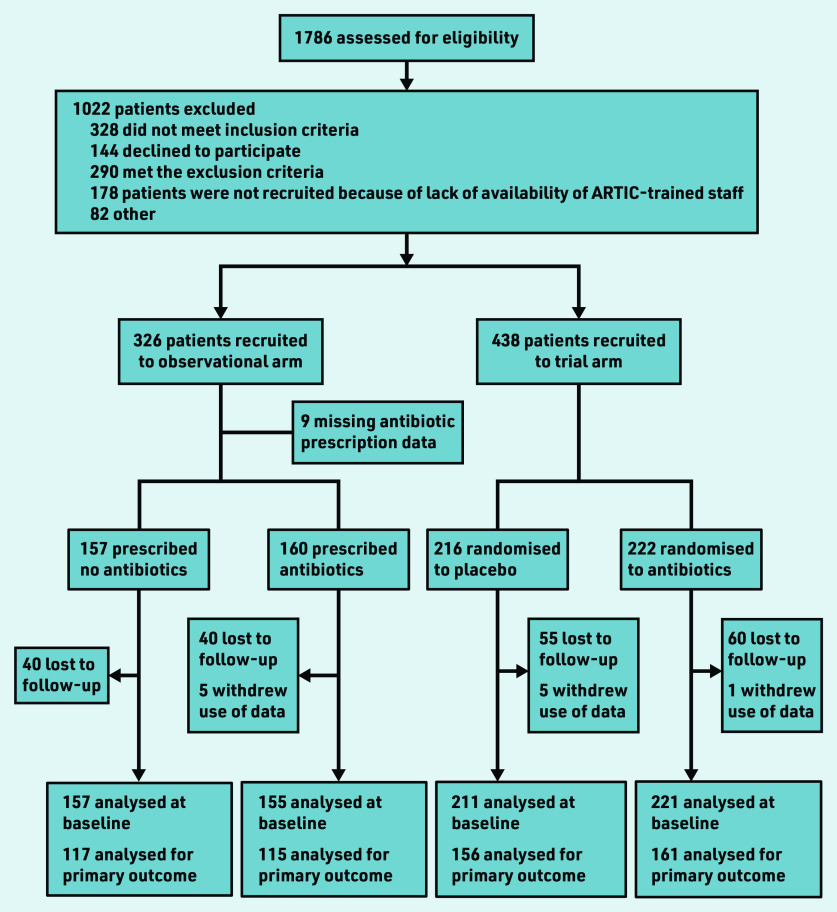
*Flow of patients through the observational study and trial.*

In total, 326 patients were recruited to the observational study, 312 with antibiotic prescription data (Figure 1). Of these 312, 157 received no antibiotic, 141 immediate antibiotic, and 14 delayed antibiotic. As the numbers with a delayed prescription were so small, these were combined with the immediate antibiotic group data for the purposes of analysis. Combined with the trial data, there were 744 participants in total, of whom 368 received no antibiotic and 376 were given or were prescribed an antibiotic.

In the observational cohort, 52/312 (16.7%) were recruited via accident and emergency (A&E)/paediatric assessment versus 260/312 (83.3%) via GP practices. In the trial, 5/432 (1.1%) were recruited via A&E/paediatric assessment versus 427/432 (98.8%) via practices.

### Proportions followed up for key outcome measures

In the observational study, the duration of illness and illness severity in days 2–4 following the consultation were recorded for 232/312 (74.4%) participants. Re-consultation was available for 271/312 (86.9%), progression of illness for 290/312 (92.9%), and side effects for 228/312 (73.1%). In the combined data, 549/744 (73.8%) reported the duration and severity of illness. Re-consultation was recorded for 672/744 (90.3%), progression of illness for 705/744 (94.8%) (Supplementary Table S1), and side effects for 538/744 (72.3%).

### Clinical characteristics

As expected, the number of children in the observational cohort with more severe clinical features (Table 1) was greater in the antibiotic group compared with the no antibiotic group — with more severe average baseline symptom scores (scores of 1.8 and 1.5, respectively), and more with abnormal chest signs (81% [126/155] and 24% [38/157], respectively), sputum production (87% [135/155] and 70% [108/155], respectively), history of fever (91% [141/155] and 64% [100/157], respectively), feeling unwell (81% [125/155] and 51% [79/155], respectively), shortness of breath (70% [109/155] and 36% [57/157], respectively), and low oxygen saturation (21% [28/132] and 7% [7/106], respectively).

**Table 1.** Baseline characteristics of observational participants and combined dataset^a^

**Characteristic**	**Observational study**		**Trial only**		**Combined**	
		
	**No antibiotics (*n* = 157)**	**Antibiotics (*n* = 155)**	**Placebo (*n* = 211)**	**Antibiotics (*n* = 221)**	**No antibiotics^b^ (*n* = 368)**	**Antibiotics (*n* = 376)**
**Male, *n*(%)**	82 (52.2)	86 (55.5)	112 (53.1)	121 (54.8)	194 (52.7)	207 (55.1)

**Age, years, median (IQR)**	3.0 (1.4–4.9)	3.1 (1.8–5.2)	3.1 (1.4–5.6)	3.2 (1.7–5.8)	3.1 (1.4–5.4)	3.2 (1.7–5.5)

**Comorbidity,^c^ *n*(%)**	17 (10.8)	18 (11.6)	31 (14.7)	24 (10.9)	48 (13.0)	42 (11.2)

**Asthma, *n*(%)**	9 (5.7)	10 (6.5)	19 (9.0)	13 (5.9)	28 (7.6)	23 (6.1)

**Long-term illness,^d^ *n*(%)**	12 (11.8)	7 (7.5)	7 (6.3)	13 (10.7)	19 (8.9)	20 (9.3)

**Baseline severity,^e^ mean (SD)**	1.5 (0.3)	1.8 (0.4)	1.6 (0.3)	1.6 (0.3)	1.6 (0.3)	1.7 (0.3)

**Prior duration of illness, days, median (IQR)**	5 (3–7)	4 (2–7)	6 (3–10)	5 (3–10)	6 (3–10)	5 (3–8)

**Abnormal chest signs,^f^ *n*(%)**	38 (24.2)	126 (81.3)	72 (34.1)	78 (35.3)	110 (29.9)	204 (54.3)

**Sputum/rattly chest, *n*(%)**	108 (69.7)	135 (87.1)	155 (73.8)	170 (77.6)	263 (72.1)	305 (81.6)

**Fever during illness, *n*(%)**	100 (63.7)	141 (91.0)	161 (76.3)	177 (80.1)	261 (70.9)	318 (84.6)

**Unwell, *n*(%)**	79 (51.0)	125 (80.7)	141 (66.8)	143 (64.7)	220 (60.1)	268 (71.3)

**Shortness of breath, *n*(%)**	57 (36.3)	109 (70.3)	95 (45.0)	104 (47.1)	152 (41.3)	213 (56.7)

**Oxygen saturation low, *n*(%)**	7 (6.6)	28 (21.2)	9 (5.4)	13 (7.7)	16 (5.9)	41 (13.6)

**STARWAVe,^g^ *n*(%)**						
Very low risk	94 (59.9)	60 (38.7)	110 (52.1)	123 (55.7)	204 (55.4)	183 (48.7)
Normal risk	60 (38.2)	77 (49.7)	95 (45.0)	94 (42.5)	155 (42.1)	171 (45.5)
High risk	3 (1.9)	18 (11.6)	6 (2.8)	4 (1.8)	9 (2.5)	22 (5.9)

**Physician rating unwell,^h^ mean (SD)**	4.9 (1.9)	6.3 (1.6)	5.5 (1.7)	5.5 (1.6)	5.3 (1.8)	5.9 (1.7)

**Parent rating of unwell,^h^ mean (SD)**	3.3 (1.6)	5.3 (1.7)	3.8 (1.7)	3.7 (1.7)	3.6 (1.7)	4.3 (1.8)

**Temperature, mean (SD)**	37.1 (0.7)	37.5 (0.9)	37.3 (0.8)	37.2 (0.8)	37.2 (0.8)	37.3 (0.8)

**Oxygen saturation, mean (SD)**	97.6 (1.5)	96.1 (2.3)	97.3 (1.6)	97.3 (1.6)	97.4 (1.6)	96.8 (2.0)

**Heart rate (beats/min), mean (SD)**	110.8 (19.0)	124.5 (21.3)	112.0 (20.3)	111.8 (17.9)	111.6 (19.8)	117.1 (20.3)

**Respiratory rate (breaths/min), mean (SD)**	24.0 (7.4)	30.7 (10.3)	24.8 (6.8)	25.4 (7.1)	24.4 (7.0)	27.6 (8.9)

**Capillary refill** >**3 s, *n*(%)**	1 (0.7)	3 (2.0)	3 (1.5)	2 (0.9)	4 (1.1)	5 (1.4)

**Consciousness, *n* (%)**						
Normal	154 (98.7)	138 (90.2)	203 (96.2)	214 (97.3)	357 (97.3)	352 (94.4)
Irritable	1 (0.6)	11 (7.2)	8 (3.8)	5 (2.3)	9 (2.5)	16 (4.3)
Drowsy	1 (0.6)	4 (2.6)	0 (0.0)	1 (0.5)	1 (0.3)	5 (1.3)

**Ill appearance, *n*(%)**	17 (10.8)	71 (45.8)	48 (22.9)	47 (21.3)	65 (17.7)	118 (31.4)

a *Missing data result in different denominators for some variables.*

b *No antibiotics for the combined dataset comprises no antibiotics for the observational data and placebo for the trial data.*

c *Comorbidity includes asthma, heart disease, renal disease, diabetes, cystic fibrosis, immunocompromised, and other chronic disease.*

d *Longer-term illness was a self-report item in the diary to the question ‘Does he/she have any long-term illness, health problem, or illness/disease which limits his/her daily activities’.*

e *Baseline severity on a scale 1 to 4: 1, none; 2, mild; 3, moderate; 4, severe.*

f *Abnormal chest signs include wheeze, stridor, grunting, nasal flaring, inter/subcostal recession, crackles/crepitations, bronchial breathing.*

g *STARWAVe prediction rule for hospital admission (Short illness, Temperature, Age, Recession, Wheeze, Asthma, Vomiting).*

h *Physician and parent rating of unwell on a scale 0 to 10. IQR = interquartile range. SD = standard deviation.*

### Propensity scores

**Figure 2.**
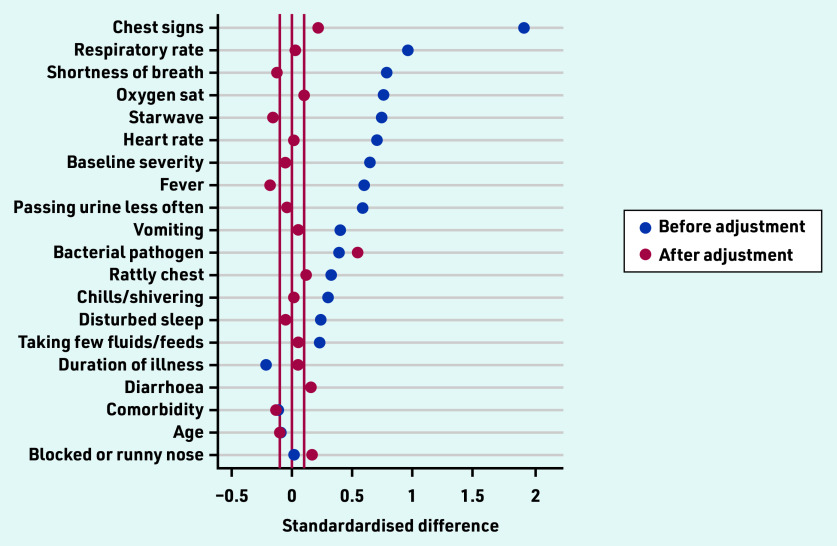
*Standardised differences in baseline characteristics between antibiotic and no antibiotic groups in the observational dataset, and after adjusting for confounding by indication using the propensity score.* *Sat = saturation. Starwave = STARWAVe prediction rule for hospital admission (Short illness, Temperature, Age, Recession, Wheeze, Asthma, Vomiting).*

The differences between antibiotic and non-antibiotic groups are shown in Figure 2 before and after adjustment using propensity scores, which demonstrates that, although there was a major impact of adjustment, there is nevertheless likely to be some residual confounding.

### Primary and secondary outcomes

For the whole cohort (combined trial and observational datasets), the crude data for the primary outcome were very skewed (no antibiotics mean 9.7 days [SD 8.1]; antibiotics 8.1 days [SD 7.5]), hence the median (interquartile range [IQR]) was used as the best summary of the data (6 [IQR 4–12] and 5 [IQR 3–9], respectively; Table 2). With antibiotics there was a non-significant reduction of approximately 1 day in duration of symptoms rated moderately bad or worse for the whole cohort (hazard ratio [HR] 1.16, 95% confidence interval [CI] = 0.95 to 1.41) (Table 2), similar to the trial alone (HR 1.13, 95% CI = 0.90 to 1.43).

**Table 2.** Primary and secondary outcomes^a,b^

**Outcome**	**Observational study only**			**Combined trial and observational data**		

	**No antibiotics**	**Antibiotics**	**Adjusted treatment estimate**	**No antibiotics**	**Antibiotics**	**Adjusted treatment estimate**
**Primary outcome**						
Duration of moderately bad or worse symptoms in days, median (IQR)	6 (4 10)	5 (3 7)	1.23 (0.83 to 1.82)	6 (4 12)	5 (3 9)	1.16 (0.95 to 1.41)

**Secondary outcome**						
Symptom severity, days 2–4,^c^ mean (SD)	1.6 (0.99)	1.9 (1.12)	0.27 ( 0.12 to 0.67)	1.8 (1.11)	1.8 (1.08)	0.14 ( 0.34 to 0.07)
Duration of symptoms until very little/no problem in days, median (IQR)	8 (5 14)	6 (5 11)	1.33 (0.88 to 2.00)	8 (5 17)	7 (5 14)	1.16 (0.95 to 1.51)
Return with new or worsening symptoms, *n*/*N* (%)	45/142 (31.7)	43/129 (33.3)	1.10 (0.53 to 2.32)	121/341 (35.5)	103/331 (31.1)	0.79 (0.55 to 1.13)
Progression of illness requiring hospital attendance or admission, *n*/*N* (%)	5/150 (3.3)	15/140 (10.7)	1.79 (0.37 to 8.57)	9/354 (2.5)	20/351 (5.7)	1.64 (0.68 to 3.95)
Side effects, *n*/*N* (%)	37/114 (32.5)	58/114 (50.9)	**3.11 (1.38 to 7.03)**	89/267 (33.3)	118/271 (43.5)	**1.62 (1.08 to 2.43)**

a *Results in bold are significant.*

b *The duration variables are summarised using hazard ratios; symptom severity is summarised using a mean difference; return with new symptoms, progression of illness, and side effects are summarised with odds ratios.*

c *Symptom severity on a scale of 0 to 6, where 0, no problem; 1, very little problem; 2, slight problem; 3, moderately bad; 4, bad; 5, very bad; 6, as bad as it could be. CI = confidence interval. IQR = interquartile range. SD = standard deviation.*

The effect of antibiotic treatment on secondary outcomes was also non-significant. The apparent non-significant increase in the progression of illness in the antibiotic group is very likely to be because of inadequate control of confounding by indication. The only outcome for which there was a statistically significant difference comparing antibiotic and no antibiotic groups was side effects, which were higher in the antibiotic group (risk ratio 1.62, 95% CI = 1.08 to 2.43).

### Subgroups

After controlling for confounding with propensity scores, none of the pre-specified subgroups had statistically significant interaction terms in either the observational dataset or in the combined dataset (Table 3). When selecting each subgroup, a suggestion of benefit was found among both children with productive sputum and children with fever, although this did not reach statistical significance.

**Table 3.** Subgroup estimates of the effectiveness of antibiotics for the duration of symptoms rated moderately bad or worse, using the combined observational and trial datasets

**Subgroup**	**Number**	**No antibiotics, median (IQR)**	**Antibiotics, median (IQR)**	**Interaction term HR (99% CI)^a^**	**Unadjusted HR (99% CI)**	**Adjusted HR^b^ (99% CI)a**
**Abnormal chest signs**				1.02 (0.68 to 1.52)		
Yes	314	5 (4–14)	5 (3–8)		1.10 (0.80 to 1.51)	1.06 (0.77 to 1.46)
No	430	7 (5–12)	5 (3–10)		1.23 (0.95 to 1.58)	1.19 (0.94 to 1.52)

**Sputum**				1.46 (0.91 to 2.34)		
Yes	568	7 (4–14)	5 (3–9)		1.34 (1.07 to 1.67)	1.29 (1.03 to 1.61)
No	171	5 (3–10)	5 (3–9)		0.85 (0.54 to 1.34)	0.86 (0.54 to 1.38)

**Fever**				1.49 (0.90 to 2.44)		
Yes	579	6 (4–13)	5 (3–8)		1.32 (1.00 to 1.77)	1.28 (1.03 to 1.60)
No	165	6 (4–12)	6 (3–23)		0.87 (0.55 to 1.39)	0.72 (0.44 to 1.18)

**Physician rating of unwell**				1.24 (0.82 to 1.86)		
Yes	488	6 (4–11)	5 (3–8)		1.32 (1.03 to 1.71)	1.26 (0.98 to 1.63)
No	254	7 (4–14)	5 (3–14)		1.04 (0.74 to 1.45)	1.03 (0.74 to 1.47)

**Shortness of breath**				0.97 (0.66 to 1.42)		
Yes	365	6 (4–11)	5 (3–9)		1.29 (0.97 to 1.71)	1.26 (0.94 to 1.68)
No	379	6 (4–14)	5 (4–10)		1.21 (0.91 to 1.59)	1.20 (0.90 to 1.59)

**Oxygen saturation <95%**				1.03 (0.51 to 2.10)		
Yes	57	6 (4.5–14.5)	6 (3–10)		0.96 (0.33 to 2.75)	1.20 (0.24 to 5.93)
No	516	6 (4–13)	5 (3–9)		1.20 (0.89 to 1.62)	1.16 (0.94 to 1.43)

**STARWAVe^c^**						
Very low risk	387	6 (4–13.5)	5 (3–9)	Reference	1.26 (0.96 to 1.65)	1.21 (0.92 to 1.59)
Normal risk	326	6 (4–11)	5 (3–8)	1.03 (0.70 to 1.52)	1.26 (0.93 to 1.72)	1.20 (0.88 to 1.64)
High risk	31	5 (3–20)	6.5 (4–11)	—	—	—

a *95% CIs for abnormal chest sign subgroup; 99% CIs for all other subgroups (99% CIs were used as these are secondary analyses).*

b *Adjusted for propensity score.*

c *STARWAVe prediction rule for hospital admission (Short illness, Temperature, Age, Recession, Wheeze, Asthma, Vomiting). CI = confidence interval. HR = hazard ratio. IQR = interquartile range.*

## DISCUSSION

### Summary

This cohort provides evidence of the limited effectiveness of antibiotics for children presenting with chest infections in primary care, even in an unwell sample of children.

### Strengths and limitations

This cohort of unwell children provides the best evidence to date of the impact of antibiotics on chest infections in children by nesting trial data (which can be limited by external validity and substantially greater drug compliance compared with observational studies^21^) within an overall cohort as this increases both the power of the analyses and generalisability.

The method of controlling for confounding by indication needed to be adapted to improve the balance between groups, and there was evidence of some residual confounding by indication for some of the outcomes — particularly for the progression of illness. It is also likely that residual confounding by indication contributed to the much higher apparent ‘side effects’ seen in the antibiotic group as it is known that diarrhoea, vomiting, and skin rash occur commonly as part of the illness for both children and adults;^36^ thus, the more common ‘side effects’ in the antibiotic group in the observational data may reflect illness severity rather than side effects *per se*.

Some potential confounders may not have been measured, for example, how rapidly a child had become ill — although prior illness duration is a reasonable proxy in acute infections.^37^ It is also possible that there is greater attribution and monitoring of known side effects when parents know their child is getting antibiotics. The differences in clinical characteristics between observational and trial datasets cannot be attributed just to clinical decision making as the range of primary care settings were different: some of the observational patients came from primary care sites that were less typical of routine general practice, and that were not able to recruit to the trial (for example, A&E).

### Comparison with existing literature

Children given antibiotics in the observational study had more severe clinical presentations than children not given antibiotics — matching the same trends in the much larger STARWAVe cohort.^38^^,^^39^ The children in the current trial cohort were also more severely affected than the children in the STARWAVe cohort, and that trend is even more apparent in the children contributing to the observational data — among children given antibiotics very high percentages had sputum production (87% compared with 63% in the STARWAVe cohort), fever (91% and 75%, respectively), and shortness of breath (70% and 46%, respectively).

Despite major differences in the clinical presentation between children given or not given antibiotics, in the current study it was found that when controlling for the propensity to prescribe antibiotics the main outcomes for the combined trial and observational data were very similar to the ‘pure’ trial data. An HR of 1.2 represents around 1 day’s difference because of antibiotics, so a hazard ratio of 1.16 is on average <1 day’s benefit from antibiotics. For the subgroup analyses, although there was some suggestive evidence of differences for some subgroups, the interaction terms were not statistically significant. For children with productive sputum or fever, the lower CIs for the HRs were only just above the null, so it is possible these are chance findings. The estimates of benefit for both the above subgroups were also not very important clinically (neither subgroup had a difference in symptom duration of >2 days). If, as the current study suggests, antibiotics are not effective this may be in part because of antibiotics not working for infections caused predominantly by viruses — but the authors of the current study in a previous publication have shown that bacterial infections were common in this cohort, and that the presence of bacteria did not predict benefit from antibiotics.^40^ It may also be in part that the time course of the inflammatory process is more important, or possibly antibiotic resistance.

### Implications for research and practice

The findings of this cohort suggest little benefit from antibiotics, even in an unwell sample of children. GPs should negotiate symptomatic management for children presenting with uncomplicated chest infections, combined with clear guidance about when any repeat consultation might be needed, and minimise the prescription of antibiotics. Future research to identify those with worse prognosis could allow a greater focus on non-antibiotic strategies and/or the need for clinical review.

## Data

De-identified participant data are available for further analyses. Request for data, with justification, should be made to the corresponding author.^41^
